# Using a Geographic Information System to Improve Childhood Lead-Screening Efforts

**DOI:** 10.5888/pcd10.120273

**Published:** 2013-06-13

**Authors:** Robert Graff

## Abstract

The Idaho Division of Public Health conducted a pilot study to produce a lead-exposure–risk map to help local and state agencies better target childhood lead-screening efforts. Priority lead-screening areas, at the block group level, were created by using county tax assessor data and geographic information system software. A series of maps were produced, indicating childhood lead-screening prevalence in areas in which there was high potential for exposure to lead. These maps could enable development of more systematically targeted and cost-effective childhood lead-screening efforts.

## Objective

Idaho does not have a funded childhood lead-poisoning prevention program. As a result, scant data are available on lead levels in Idaho children, and resources for screening for lead poisoning are limited. Although some screening is done, it is unknown whether children at highest risk of exposure to lead-based paint, the leading cause of childhood lead poisoning (1), are the ones being screened. To help local and state agencies better target childhood lead-screening efforts to the highest risk geographic areas, the Idaho Division of Public Health (DPH) conducted a pilot study to produce a lead-exposure–risk map for Ada County, the most populous county in Idaho.

## Methods

**Figure 1:**
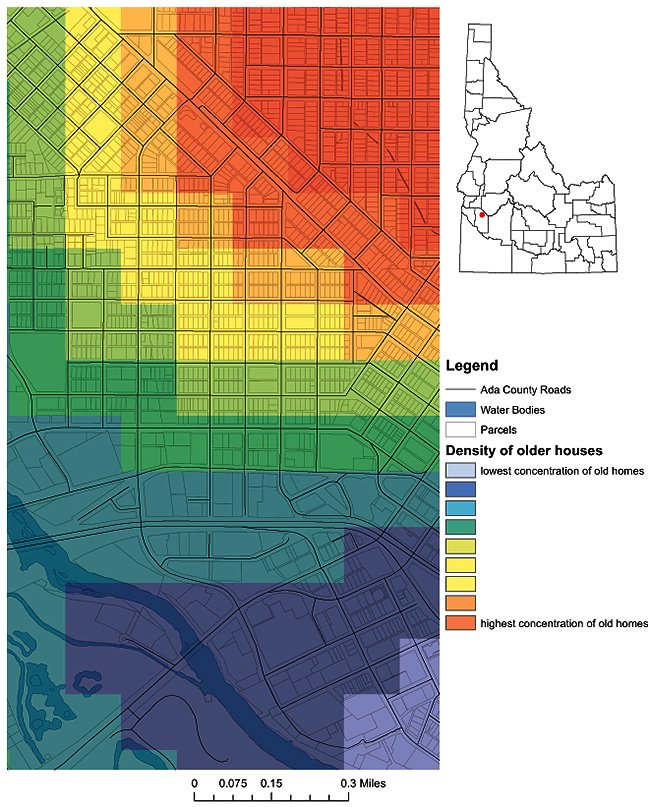
A first step in understanding paint-based lead exposure risk in Ada County, Idaho. By using county tax assessor data, a point density map was created to identify hot-spots of housing structures built before 1950.

The rural nature of states like Idaho often means working with fewer data sources. Therefore, the lead-exposure risk map had to be constructed with scant clinical data in the absence of universal lead reporting and, apart from Medicaid data, without a denominator for lead-screenings. The key piece of information for this map was parcel-level data (age of housing structure) from the county tax assessor’s office. Data were obtained in November 2011, and analysis for the project began in March 2012. We used a geographic information system (GIS), ArcGIS 10.0 (Esri, Redlands, California), to identify potential exposure sources and high-risk areas. Parcel data were imported into ArcGIS 10.0, and housing structures were categorized by year built, from highest to lowest risk: built in or before 1950 when lead paint contained almost 50% lead by dry weight (2), built from 1951 through 1978, and built from 1979 to the time of our study. We used these categories to create a heat map depicting parcel-level, lead-based paint exposure risk (Figure 1). Block-group–level median household income data (3) was joined to the parcel data as a proxy variable for the condition of the home. A median household income of $50,000 was used as a cut-off point; based on previous Idaho DPH analysis, a household income of less than $50,000 was assumed to confer a slightly higher risk of lead exposure (4).

**Figure 2:**
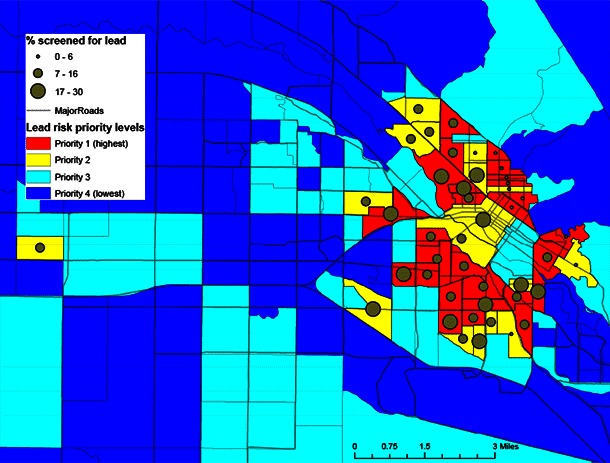
Based on household income and housing age variables, census block groups indicate the potential risk of exposure to lead-based paint. Geocoded Medicaid enrollment data specify the percentage of children screened for lead in areas with high potential for exposure (3,5,6).

To create a simple and replicable method of identifying high-risk areas, parcel data were aggregated to the block-group level by using the year-built risk categories described above (ie, number and percentage of pre-1950 homes per block group). For modeling, a 9-point weighting system was used to assign block groups on the basis of the median household income cut-off point, the number of homes in each year-built risk category, and the overall percentage of homes built pre-1950. After several runs, final model weights were validated against the known distributions of houses within block groups. The model gave greatest weight to older houses and generated a 4-tier scale of decreasing exposure risk by block group: Priority 1 (7 to 9 on the weighting scale), Priority 2 (5 to 6), Priority 3 (3 to 4), and Priority 4 (1 to 2). Current enrollment data, as of August 2012, for children on Medicaid aged 0 to 6 years were spatially joined to the block group layer. Use of this data was approved by the Idaho Division of Public Health’s institutional review board and contained dates of lead-screening but not testing levels. These data allowed for analysis of how many Medicaid-enrolled children residing in priority block groups were being screened for lead (Figure 2).

## Results

A primary outcome of the pilot study was identification of specific residential areas (block groups) in which childhood lead screening should be a priority because of the high risk of exposure to lead-based paint. Ada County has 169 block groups; 27 (16.0%) of its block groups were in the Priority 1 category, and 17 (10.0%) block groups were in the Priority 2 category. In 2010, 10.3% of Ada County’s population was aged 0 to 6 years, and 15.4% of these children were living in the 44 high-priority block groups. The highest priority block groups were clustered in the city center of Boise, the county seat of Ada County. Most residences built before 1950 are in this area. Merging Medicaid childhood screening data with the priority block groups confirmed that 78.0% of children at high risk of lead exposure were not being screened.

## Discussion

The use of GIS to assess childhood lead-poisoning risk has long been established (7). Several researchers have used GIS to create replicable models for identifying potential exposure sources and high risk areas (8–11). Our study combined parcel and Medicaid screening data, which allowed for identification of risk areas while retaining finer resolution data of exposure risks within block groups. Mapping potential lead exposure is a necessary step toward moving children’s environmental-health interventions from mitigative to preventive (12). Through the identification of geographic areas with the highest risk of exposure to lead-based paint, maps produced from this project have moved Medicaid closer to identifying at-risk children before they become symptomatic.

Convincing physicians that lead exposure is an issue in their Medicaid patient population is an ongoing challenge. Maps designed on the basis of the age of house structures might more readily convey risk information. Efforts are currently under way to use GIS software to identify physicians with high numbers of child patients living in priority screening areas to enable Medicaid to work with specific physicians and provide them with the necessary tools (eg, lead analyzers) to screen more of their at-risk children. Additionally, future potential exists to conduct direct education with parents of newborn children residing in high-risk areas via a linkage with birth certificate data (13).

A limitation of our study was that the extent to which the priority areas our maps identify represent an increased number of childhood lead-poisoning cases has yet to be assessed. Such an assessment could be facilitated in the future through universal blood lead level reporting requirements. Anecdotal evidence suggests physicians may screen for lead but opt to bill this service under a more general physical examination code that has higher Medicaid reimbursement. As a result, Medicaid lead-screening estimates may underestimate actual screening levels. Future research is needed to determine the extent of this underestimation.

This mapping project has enhanced the relationship between Medicaid and programs in the Idaho DPH and has led to new ideas regarding lead screening. We anticipate that the methods used to create these maps can be readily replicated, ultimately resulting in the development of more systematically targeted and cost-effective childhood lead-screening efforts by local and state agencies.
